# Risk stratification for the detection of metachronous polyps after bowel screening polypectomy: clinical outcomes from the Integrated Technologies for Improved Polyp Surveillance (INCISE) study cohort

**DOI:** 10.1093/bjsopen/zrad034

**Published:** 2023-05-09

**Authors:** Mark S Johnstone, Reiss Stoops, Gerard Lynch, Jennifer Hay, Jakub Jawny, William Sloan, Joanne Edwards, Stephen T McSorley

**Affiliations:** Academic Unit of Surgery, School of Medicine, University of Glasgow, Glasgow, UK; Academic Unit of Surgery, School of Medicine, University of Glasgow, Glasgow, UK; Wolfson Wohl Cancer Research Centre, School of Cancer Sciences, University of Glasgow, Glasgow, UK; Glasgow Tissue Research Facility, School of Cancer Sciences, University of Glasgow, Glasgow, UK; Department of Pathology, Queen Elizabeth University Hospital, Glasgow, UK; Research and Development, Queen Elizabeth University Hospital, Glasgow, UK; Wolfson Wohl Cancer Research Centre, School of Cancer Sciences, University of Glasgow, Glasgow, UK; Academic Unit of Surgery, School of Medicine, University of Glasgow, Glasgow, UK

## Abstract

**Background:**

After colorectal polypectomy, 20–50 per cent of patients develop metachronous polyps and some have increased colorectal cancer risk. British Society of Gastroenterology (BSG) 2020 guidelines recommend surveillance colonoscopy for high-risk patients based on index pathology. The aim of this study was to evaluate metachronous lesion outcome using BSG 2020 criteria.

**Methods:**

A retrospective, multicentred study was conducted including patients who had polypectomy during screening colonoscopy (2009–2016) followed by surveillance. Demographics, index pathology, and BSG 2020 risk criteria were compared with regard to metachronous lesion pathology (non-advanced *versus* advanced lesions) and timing of detection (early *versus* late). Advanced lesions were defined as adenomas/serrated polyps greater than or equal to 10 mm, high-grade dysplasia, serrated polyps with dysplasia, or colorectal cancer, and late lesions those detected greater than 2 years after the index procedure.

**Results:**

Of 3090 eligible patients, 2643 were included. Among these, retrospective BSG 2020 application would have excluded 51.5 per cent from surveillance. After a median of 36 months, the advanced polyp/colorectal cancer rate in BSG 2020 high-risk patients was 16.3 *versus* 13.0 per cent in low-risk patients. Older age (*P* = 0.008) correlated with advanced metachronous lesions. Male sex, greater than five polyps, and BSG 2020 high-risk criteria correlated with non-advanced and advanced lesions (*P* < 0.001). Older age (*P* < 0.001), villous features (*P* = 0.006), advanced index polyp (*P* = 0.020), and greater than five polyps (*P* < 0.001) correlated with early metachronous lesions. Male sex and BSG 2020 high-risk criteria correlated with early and late lesions (*P* < 0.001). On multivariable regression, increased polyp number (odds ratio (OR) 1.15 (95 per cent c.i. 1.07 to 1.25); *P* < 0.001) and villous features (OR 1.49 (95 per cent c.i. 1.05 to 2.10); *P* = 0.025) independently correlated with early advanced lesions. The rate of non-advanced and advanced metachronous polyps was higher in BSG 2020 high- *versus* low-risk patients (44.4 *versus* 35.4 per cent for non-advanced and 15.7 *versus* 11.8 per cent for advanced; *P* < 0.001), but the colorectal cancer rate was similar (0.6 *versus* 1.2 per cent). However, when examining only lesions detected greater than 2 years after the index colonoscopy in high- *versus* low-risk patients, no significant differences were observed (*P* = 0.140).

**Conclusion:**

BSG 2020 criteria correlated with metachronous polyps, but did not differentiate advanced and non-advanced lesions and were not predictive of late lesions.

## Teaser

This retrospective, multicentred study of 2643 patients undergoing surveillance after polypectomy at a screening colonoscopy compared outcomes using British Society of Gastroenterology 2020 risk criteria. The advanced metachronous polyp/colorectal cancer rate in British Society of Gastroenterology 2020 high-risk patients was 16.3 per cent *versus* 13.0 per cent in low-risk patients. Post-polypectomy risk stratification for surveillance may benefit from refinement.

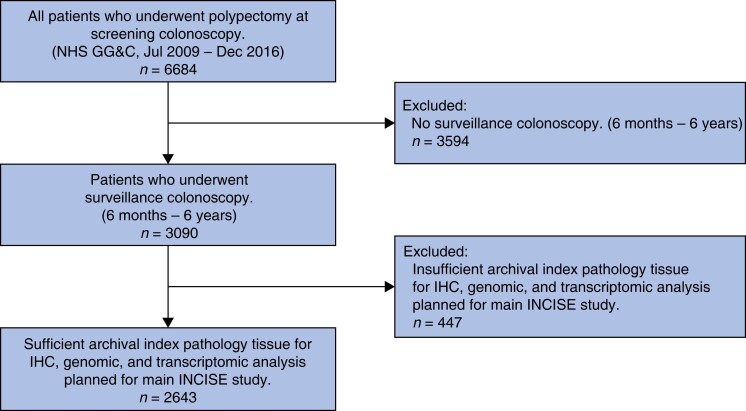

## Introduction

Colorectal cancer (CRC) is the fourth most common cancer in the UK, with approximately 43 000 new cases and 16 500 deaths each year^1^. CRC develops from precursor, benign colorectal polyps (both adenomas and serrated polyps)^2–5^. Such premalignant polyps are common and, as malignant transformation takes 7–15 years, represent excellent targets for cancer prevention by polypectomy; by excising benign, dysplastic polyps endoscopically prior to malignant transformation, CRC incidence can be reduced^6,7^. In addition to early cancer detection, the Bowel Cancer Screening Programme aims to identify benign, dysplastic polyps and remove them before malignant transformation. However, after polypectomy, it is estimated that 20–50 per cent of patients will develop further, metachronous polyps^8^ and a proportion are at higher long-term risk of developing CRC^9^. Therefore, a subset of patients are invited to return for a surveillance colonoscopy^5,7^. It is unsustainable to offer surveillance colonoscopy to all. Additionally, patients found to have very low-risk lesions at the index colonoscopy are unlikely to benefit from undergoing a further invasive colonoscopy. Instead, patients are stratified for risk based on polyp histology, grade of dysplasia, size, and number, according to the latest British Society of Gastroenterology (BSG)/Association of Coloproctology of Great Britain and Ireland/Public Health England post-polypectomy surveillance 2020 guidelines^5^. On this basis, patients are divided into high- and low-risk groups; high-risk patients are invited to a surveillance colonoscopy at 3 years and low-risk patients are discharged to the screening programme^5^. Although the number of patients qualifying for surveillance with these conventional risk measures is limited, surveillance colonoscopy still accounts for 100 000 of the 700 000 colonoscopies performed in England each year^10^. A more accurate risk stratification would allow for more efficient NHS resource allocation.

The Integrated Technologies for Improved Polyp Surveillance (INCISE) project is a large, retrospective, multi-partner collaborative study that aims to use patient characteristics, digital pathology, immunohistochemistry (IHC), and genomic and transcriptomic features of index polyp tissue to predict future polyp risk and refine current surveillance protocols^11^. The aim of this study was to retrospectively apply the BSG 2020 guidelines to the INCISE cohort and compare metachronous polyp/CRC rate using BSG 2020 high- and low-risk features.

## Methods

### Study design

A retrospective, multicentred observational cohort study was performed. The formation of the INCISE cohort has previously been described^12^. Briefly, a prospectively maintained cohort of all patients participating in the first round of the Scottish Bowel Screening Programme in NHS Greater Glasgow & Clyde (GG&C) was reviewed. Patients who underwent polypectomy at a screening colonoscopy in NHS GG&C between May 2009 and December 2016 were considered for inclusion. During the study interval, the Scottish Bowel Screening Programme was based on a biannual guaiac faecal occult blood test (gFOBT), followed by invitation to a colonoscopy for those with a positive stool test^13^. Patients were only included if they had a histologically confirmed premalignant polyp (adenoma or serrated polyp, excluding diminutive rectal hyperplastic polyps less than 5 mm) at their index screening colonoscopy. Patients must have undergone a further colonoscopy 6 months to 6 years after their index endoscopy, to allow identification of those patients who went on to develop metachronous polyps or CRC. Patients were excluded if they were found to have CRC at their index screening colonoscopy, had a previous histological diagnosis of CRC, had a diagnosis of inflammatory bowel disease, had a known inherited polyposis or CRC syndrome, or did not have a surveillance colonoscopy within the above date ranges. Each patient was assigned a unique INCISE number and the entire, anonymized database was stored on the NHS Safe Haven platform (Safe Haven, NHS Scotland) to ensure compliance with data protection and patient confidentiality. Ethics approval was obtained for the INCISE project (GSH/20/CO/002), which was reported according to STROBE guidelines^14^.

### Variables and data sources

**Fig. 1:**
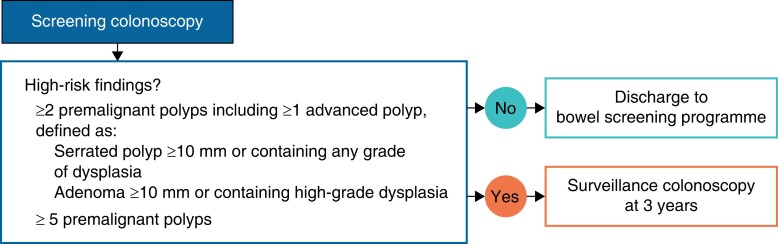
British Society of Gastroenterology and Association of Coloproctology of Great Britain and Ireland post-polypectomy surveillance guidelines

Patient demographics, co-morbidities, and medications were extracted by searching local electronic case notes with the unique Scottish community health index (CHI) number used as the linkage variable. Demographics collected included age, sex, and deprivation. Deprivation was quantified using the Scottish Index of Multiple Deprivation (SIMD) score, which is a measure of an area’s deprivation according to income, employment, education, health, access to services, crime, and housing^15^. Co-morbidities were recorded individually and used to calculate the Charlson co-morbidity index^16^. The local pathology database was used to determine the number of index polypectomy specimens and the histological subtype (adenoma *versus* serrated polyp), location (rectum, left-sided colonic, and right-sided colonic), size, morphology (presence or absence of villous architecture), and degree of dysplasia (high- or low-grade) of the most advanced index polyp. The latest version of the BSG 2020 guidelines^5^ was used to define those patients with a non-advanced index polyp (adenoma less than 10 mm and not containing high-grade dysplasia (HGD) or serrated polyp less than 10 mm and not containing dysplasia), those with an advanced index polyp (adenoma greater than or equal to 10 mm or containing HGD or a serrated polyp greater than or equal to 10 mm or containing any grade of dysplasia), and those deemed at high risk of developing metachronous polyps (*Fig. 1*). To define outcomes for each patient, the electronic endoscopy reporting software (Unisoft Medical Systems GI Reporting Software) and electronic pathology database (TelePath) were used to determine the presence or absence of metachronous lesions at surveillance colonoscopy.

### Outcomes of interest

The primary study outcome was the detection of metachronous lesions (no metachronous lesions *versus* non-advanced lesions *versus* advanced lesions detected at surveillance colonoscopy). Non-advanced lesions were defined as non-advanced polyps and advanced lesions were defined as advanced polyps (as defined above) or CRC. The secondary study outcome was the detection of metachronous lesions by timing (no metachronous lesions *versus* early metachronous lesion detection *versus* late metachronous lesion detection). Early metachronous lesions were defined as those detected less than or equal to 2 years after the index polypectomy and late metachronous lesions were defined as those detected greater than 2 years after the index polypectomy.

### Statistics

Demographics including age, sex, screening cycle, deprivation, co-morbidities, medications, and index polyp characteristics and location including BSG 2020 risk categories were compared for the primary and secondary outcomes of interest using cross-tabulation and the *χ*^2^ test for categorical variables and Kruskal–Wallis one-way ANOVA for continuous data. A value of *P* < 0.050 was considered statistically significant. Multivariate polynomial regression was used to identify independent factors that correlated with advanced metachronous lesion development both less than or equal to 2 years and greater than 2 years from index colonoscopy.

## Results

### Study population

**Fig. 2:**
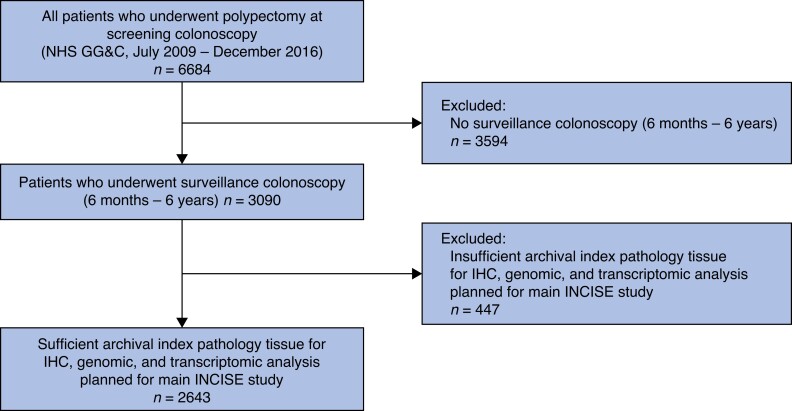
Flow chart showing formation of the Integrated Technologies for Improved Polyp Surveillance cohort NHS, National Health Service; GG&C, Greater Glasgow & Clyde; IHC, immunohistochemistry; INCISE; Integrated Technologies for Improved Polyp Surveillance.

*Figure 2* shows the INCISE cohort patient selection. Of 6684 patients who underwent polypectomy at a screening colonoscopy during the study interval, 3090 underwent surveillance colonoscopy (6 months to 6 years after the index colonoscopy) and 2643 patients were included in the final analysis. All patients in this study underwent surveillance colonoscopy, based on the guidance in use at the time^17,18^. However, applying the most recent BSG 2020 guidelines^5^ to this cohort of patients, 1360 (51.5 per cent) patients would be low risk and would no longer qualify for surveillance. The median age was 63 (range 50–83) years, with a male : female ratio of 2.2 : 1. Overall, 32.8 per cent had a single index polyp, 54.5 per cent had 2–4 polyps, and 12.6 per cent had greater than or equal to five polyps. Furthermore, 1730 (65.5 per cent) patients had a polyp greater than or equal to 10 mm found at index colonoscopy, 285 (10.8 per cent) had a polyp containing HGD, and 1038 (39.3 per cent) had a polyp with villous morphology. In total, 1757 (66.5 per cent) patients had an advanced polyp found at the index colonoscopy, of which 1693 (96.4 per cent) had an advanced adenoma and 64 (3.6 per cent) had an advanced sessile polyp.

### Outcomes of surveillance

The median time to surveillance colonoscopy was 36 (range 6–83) months. At surveillance colonoscopy, 1205 (45.6 per cent) patients had no metachronous lesion found, whereas 1438 (54.4 per cent) were found to have a metachronous lesion. A total of 1051 (39.8 per cent) patients had non-advanced polyps, 363 (13.7 per cent) had advanced polyps, and 24 (0.9 per cent) patients were found to have CRC. Of these 1438 patients, 655 (45.5 per cent) had their lesions identified early (within 2 years of the index colonoscopy), and 783 (54.5 per cent) patients had their lesions identified late (after 2 years).

### Variables associated with metachronous lesion risk

*Table 1* shows a comparison of patient demographics and index pathology characteristics between those found to have no metachronous lesions, those found to have non-advanced metachronous polyps, and those found to have advanced polyps or CRC (primary study outcome). Patients with advanced lesions at follow-up were older (no metachronous lesion *versus* non-advanced lesion *versus* advanced lesion: median age of 63, 63, and 65 years respectively; *P* = 0.008). Patients with either a non-advanced or advanced metachronous lesion were more likely to be male (no metachronous lesion *versus* non-advanced lesion *versus* advanced lesion: male 63.9, 73.8, and 71.8 per cent respectively; *P* < 0.001), to have undergone their index screening colonoscopy in the later years of the study (*P* = 0.037), to have congestive heart failure (*P* = 0.037), and to take aspirin (*P* = 0.035) or a statin (*P* = 0.004). Having an increased number of index polyps was associated with a higher risk of either non-advanced or advanced metachronous lesions (no metachronous lesion *versus* non-advanced lesion *versus* advanced lesion (greater than or equal to five polyps): 7.1, 16.7, and 18.9 per cent respectively; *P* < 0.001). As compared with having an index polyp in the rectum, right-sided colonic index polyps were associated with a higher rate of metachronous lesions and left-sided index polyps were associated with a lower risk (*P* = 0.001). The BSG 2020 guideline risk stratification of the index colonoscopy was significantly associated with metachronous lesion likelihood, but did not differentiate those with metachronous non-advanced and advanced lesions (no metachronous lesion *versus* non-advanced lesion *versus* advanced lesion: BSG 2020 high risk 41.7, 54.2, and 54.3 per cent respectively; *P* < 0.001).

**Table 1:** Factors associated with metachronous polyps or colorectal cancer after polypectomy at the index screening colonoscopy, grouped by advancement

Variable		All (*n* = 2643)	Follow-up results			*P*
			No lesions (*n* = 1205)	Non-advanced polyps (*n* = 1051)	Advanced polyps or CRC (*n* = 387)	
**Demographics (*n* = 2643)**						
Age (years)	Median (i.q.r.)	63 (57–69)	63 (57–69)	63 (59–69)	65 (59–69)	0.008
Sex	Male	1824 (69.0)	770 (63.9)	776 (73.8)	278 (71.8)	<0.001
Screening cycle	2009–2011	824 (31.2)	406 (33.7)	305 (29.0)	113 (29.2)	0.037
	2011–2013	848 (32.1)	398 (33.0)	328 (31.2)	122 (31.5)	
	2013–2015	628 (23.8)	267 (22.2)	265 (25.2)	96 (24.8)	
	2015–2017	343 (13.0)	134 (11.1)	153 (14.6)	56 (14.5)	
SIMD quintile 2009 (*n* = 2375)	1	785 (33.1)	352 (32.7)	322 (33.9)	111 (31.6)	0.288
	2	417 (17.6)	177 (16.5)	176 (18.4)	65 (18.5)	
	3	390 (16.4)	180 (16.7)	156 (16.4)	54 (15.4)	
	4	328 (13.8)	137 (12.7)	133 (14.0)	58 (16.5)	
	5	455 (19.2)	229 (21.3)	163 (17.2)	63 (17.9)	
**Co-morbidity (*n* = 2643)**						
Myocardial infarction	Yes	143 (5.4)	59 (4.9)	69 (6.6)	15 (3.9)	0.076
Congestive cardiac failure	Yes	50 (1.9)	16 (1.3)	21 (2.0)	13 (3.4)	0.037
Peripheral vascular disease	Yes	61 (2.3)	24 (2.0)	26 (2.5)	11 (2.8)	0.562
Cerebrovascular accident	Yes	83 (3.1)	36 (3.0)	30 (2.9)	17 (4.4)	0.306
Dementia	Yes	1 (0.04)	0 (0)	1 (0.1)	0 (0)	0.469
COPD	Yes	183 (6.9)	83 (6.9)	76 (7.2)	24 (6.2)	0.791
Rheumatic disease	Yes	34 (1.3)	18 (1.5)	13 (1.2)	3 (0.8)	0.542
Peptic ulcer disease	Yes	86 (3.3)	43 (3.6)	32 (3.0)	11 (2.8)	0.693
Mild liver disease	Yes	42 (1.6)	21 (1.7)	16 (1.5)	5 (1.3)	0.806
Moderate/severe liver disease	Yes	18 (0.7)	9 (0.7)	6 (0.6)	3 (0.8)	0.854
Diabetes mellitus uncomplicated	Yes	126 (4.8)	56 (4.6)	47 (4.5)	23 (5.9)	0.492
Diabetes mellitus complicated	Yes	10 (0.4)	4 (0.3)	5 (0.5)	1 (0.3)	0.786
Hemi/paraplegia	Yes	10 (0.4)	4 (0.3)	4 (0.4)	2 (1)	0.876
Renal disease	Yes	33 (1.2)	14 (1.2)	12 (1.1)	7 (2)	0.561
Any malignancy	Yes	177 (6.7)	70 (5.8)	82 (7.8)	25 (6)	0.165
Metastatic malignancy	Yes	8 (0.3)	2 (0.2)	5 (0.5)	1 (0.3)	0.404
HIV/AIDS	Yes	0 (0)	0 (0)	0 (0)	0 (0)	–
Charlson co-morbidity index (0–33)	Median (i.q.r.)	0 (0–1)	0 (0–1)	0 (0–1)	0 (0–1)	0.237
**Medication (*n* = 2472)**						
ACE-I	Yes	673 (27.2)	293 (25.9)	284 (29.0)	96 (26.7)	0.283
ARB	Yes	276 (11.2)	115 (10.2)	112 (11.4)	49 (13.6)	0.186
Aspirin	Yes	795 (32.2)	334 (29.5)	335 (34.1)	126 (35.0)	0.035
Statin	Yes	1120 (45.3)	472 (41.7)	470 (47.9)	178 (49.4)	0.004
Steroid	Yes	331 (13.4)	157 (13.9)	127 (12.9)	47 (13.1)	0.804
NSAIDs	Yes	1037 (41.9)	485 (42.9)	419 (42.7)	133 (36.9)	0.114
Immunosuppressants	Yes	62 (2.5)	34 (3.0)	17 (1.7)	11 (3.1)	0.135
Metformin	Yes	218 (8.8)	91 (8.0)	88 (9.0)	39 (10.8)	0.274
**Pathology (*n* = 2643)**						
Index polyp advanced*	Yes	1757 (67.5)	809 (67.1)	683 (65.0)	265 (68.5)	0.372
Index polyp number	1	868 (32.8)	482 (40.0)	273 (26.0)	113 (29.2)	<0.001
	2–4	1441 (54.5)	638 (52.9)	602 (57.3)	201 (51.9)	
	≥5	334 (12.6)	85 (7.1)	176 (16.7)	73 (18.9)	
Index polyp villous*	Yes	1038 (39.3)	485 (40.2)	388 (36.9)	165 (40.6)	0.092
Index polyp type*	Adenoma	2503 (94.7)	1142 (94.8)	993 (94.5)	368 (95.1)	0.891
	Serrated polyp	140 (5.3)	63 (5.2)	58 (5.5)	19 (4.9)	
Index polyp HGD*	Yes	285 (10.8)	124 (10.3)	114 (10.8)	47 (12.1)	0.590
Index polyp size* (mm)	<10	913 (34.5)	408 (33.9)	375 (35.7)	130 (33.6)	0.605
	≥10	1730 (65.5)	797 (66.1)	676 (64.3)	257 (66.4)	
Index polyp location* (*n* = 2639)	Rectum	345 (13.1)	158 (13.1)	129 (12.3)	58 (15.0)	0.001
	Left colon	1595 (60.3)	769 (63.8)	618 (58.8)	208 (53.7)	
	Right colon	697 (26.4)	275 (22.8)	302 (28.7)	120 (31.0)	
BSG 2020 risk index procedure	Low	1360 (51.5)	702 (58.3)	481 (45.8)	177 (45.7)	<0.001
	High	1283 (48.5)	503 (41.7)	570 (54.2)	210 (54.3)	

Values are *n* (%) unless otherwise indicated. CRC, colorectal cancer; SIMD, Scottish Index of Multiple Deprivation; COPD, chronic obstructive pulmonary disease; ACE-I, angiotensin converting enzyme inhibitor; ARB, angiotensin receptor blocker; NSAIDs, non-steroidal anti-inflammatory drugs; HGD, high-grade dysplasia; BSG, British Society of Gastroenterology.

*Applies to the most advanced polyp if multiple polyps removed during the index procedure.

Next, the same comparison of demographics and index pathology characteristics was made between those found to have no metachronous lesion, an early metachronous lesion (less than or equal to 2 years after the index colonoscopy), and a late metachronous lesion (greater than 2 years after the index colonoscopy) (*Table 2*). Patients who developed early metachronous lesions were significantly older (no metachronous lesion *versus* early lesion *versus* late lesion: median age 63, 65, and 63 years respectively; *P* < 0.001) and were more likely to be taking angiotensin receptor blockers (*P* = 0.001), aspirin (*P* = 0.023), or a statin (*P* = 0.002). Patients with either an early or late metachronous lesion were more likely to be male (no metachronous lesion *versus* early lesion *versus* late lesion: male 63.9, 74.2, and 72.5 per cent respectively; *P* < 0.001) and to have congestive heart failure (*P* = 0.015). Having an index advanced polyp was associated with a higher risk of early, but not late, metachronous lesions (no metachronous lesion *versus* early lesion *versus* late lesion: advanced index polyp 67.1, 69.6, and 62.8 per cent respectively; *P* = 0.020). Having an increased number of index polyps was associated with early or late metachronous lesions (no metachronous lesion *versus* early lesion *versus* late lesion (greater than or equal to five polyps):7.1, 25.2, and 10.7 per cent respectively; *P* < 0.001). Index villous lesions were associated with early, but not late, metachronous lesions (*P* = 0.006). Right-sided index lesions were associated with a higher risk of both early and late metachronous lesions (*P* < 0.001). BSG 2020 high-risk features were associated with a higher rate of both early and late metachronous lesions, but there was a stronger association with early lesions (no metachronous lesion *versus* early lesion *versus* late lesion: BSG 2020 high-risk groups 41.7, 64.1, and 46.0 per cent respectively; *P* < 0.001).

**Table 2:** Factors associated with metachronous polyps or colorectal cancer after polypectomy at the index screening colonoscopy, grouped by time of detection

Variable		All (*n* = 2643)	Follow-up results			*P*
			No lesions (*n* = 1205)	Early lesions (*n* = 655)	Late lesions (*n* = 783)	
**Demographics (*n* = 2643)**						
Age (years)	Median (i.q.r.)	63 (57–69)	63 (57–69)	65 (59–71)	63 (58–69)	<0.001
Sex	Male	1824 (69.0)	770 (63.9)	486 (74.2)	568 (72.5)	<0.001
Screening cycle	2009–2011	824 (31.2)	406 (33.7)	198 (30.2)	220 (28.1)	<0.001
	2011–2013	848 (32.1)	398 (33.0)	168 (25.6)	282 (36.0)	
	2013–2015	628 (23.8)	267 (22.2)	163 (24.9)	198 (25.3)	
	2015–2017	343 (13.0)	134 (11.1)	126 (19.2)	83 (10.6)	
SIMD quintile 2009 (*n* = 2375)	1	785 (33.1)	352 (32.7)	200 (34.3)	233 (32.5)	0.185
	2	417 (17.6)	177 (16.5)	105 (18.0)	135 (18.8)	
	3	390 (16.4)	180 (16.7)	101 (17.3)	109 (15.2)	
	4	328 (13.8)	137 (12.7)	87 (14.9)	104 (14.5)	
	5	455 (19.2)	229 (21.3)	90 (15.4)	136 (19.0)	
**Co-morbidity (*n* = 2643)**						
Myocardial infarction	Yes	143 (5.4)	59 (4.9)	46 (7.0)	38 (4.9)	0.109
Congestive cardiac failure	Yes	50 (1.9)	16 (1.3)	21 (3.2)	13 (1.7)	0.015
Peripheral vascular disease	Yes	61 (2.3)	24 (2.0)	16 (2.4)	21 (2.7)	0.585
Cerebrovascular accident	Yes	83 (3.1)	36 (3.0)	24 (3.7)	23 (2.9)	0.674
Dementia	Yes	1 (0.04)	0 (0)	0 (0)	1 (0.1)	0.305
COPD	Yes	183 (6.9)	83 (6.9)	50 (7.6)	50 (6.4)	0.648
Rheumatic disease	Yes	34 (1.3)	18 (1.5)	9 (1.4)	7 (0.9)	0.497
Peptic ulcer disease	Yes	86 (3.3)	43 (3.6)	22 (3.4)	21 (2.7)	0.545
Mild liver disease	Yes	42 (1.6)	21 (1.7)	6 (0.9)	15 (1.9)	0.271
Moderate/severe liver disease	Yes	18 (1.6)	9 (0.7)	5 (0.8)	4 (0.5)	0.787
Diabetes mellitus uncomplicated	Yes	126 (4.8)	56 (4.6)	37 (5.6)	33 (4.2)	0.430
Diabetes mellitus complicated	Yes	10 (0.4)	4 (0.3)	3 (1)	3 (0.4)	0.914
Hemi/paraplegia	Yes	10 (0.4)	4 (0.3)	2 (0.3)	4 (0.5)	0.769
Renal disease	Yes	33 (1.2)	14 (1.2)	9 (1.4)	10 (1.3)	0.922
Any malignancy	Yes	177 (6.7)	70 (5.8)	51 (7.8)	56 (7.2)	0.221
Metastatic malignancy	Yes	8 (0.3)	2 (0.2)	4 (0.6)	2 (0.3)	0.239
HIV/AIDS	Yes	0 (0)	0 (0)	0 (0)	0 (0)	–
Charlson co-morbidity index (0–33)	Median (i.q.r.)	0 (0–1)	0 (0–1)	0 (0–1)	0 (0–1)	0.079
**Medication (*n* = 2472)**						
ACE-I	Yes	673 (27.2)	293 (25.9)	169 (28.0)	211 (28.6)	0.387
ARB	Yes	276 (11.2)	115 (10.2)	93 (15.4)	68 (9.2)	0.001
Aspirin	Yes	795 (32.2)	334 (29.5)	216 (35.8)	245 (33.2)	0.023
Statin	Yes	1120 (45.3)	472 (41.7)	303 (50.2)	345 (46.8)	0.002
Steroid	Yes	331 (13.4)	157 (13.9)	80 (13.2)	94 (12.8)	0.778
NSAIDs	Yes	1037 (41.9)	485 (42.9)	233 (38.6)	319 (43.3)	0.152
Immunosuppressants	Yes	62 (2.5)	34 (3.0)	11 (1.8)	17 (2.3)	0.296
Metformin	Yes	218 (8.8)	91 (8.0)	68 (11.3)	59 (8.0)	0.052
**Pathology (*n* = 2643)**						
Index polyp advanced*	Yes	1757 (67.5)	809 (67.1)	456 (69.6)	492 (62.8)	0.020
Index polyp number	1	868 (32.8)	482 (40.0)	129 (19.7)	257 (32.8)	<0.001
	2–4	1441 (54.5)	638 (52.9)	361 (55.1)	442 (56.4)	
	≥5	334 (12.6)	85 (7.1)	165 (25.2)	84 (10.7)	
Index polyp villous*	Yes	1038 (39.3)	485 (40.2)	280 (42.7)	273 (34.9)	0.006
Index polyp type*	Adenoma	2503 (94.7)	1142 (94.8)	627 (95.7)	734 (93.7)	0.244
	Serrated polyp	140 (5.3)	63 (5.2)	28 (4.3)	49 (6.3)	
Index polyp HGD*	Yes	285 (10.8)	124 (10.3)	84 (12.8)	77 (9.8)	0.144
Index polyp size* (mm)	<10	913 (34.5)	408 (33.9)	210 (32.1)	295 (37.7)	0.066
	≥10	1730 (65.5)	797 (66.1)	445 (67.9)	488 (62.3)	
Index polyp location* (*n* = 2639)	Rectum	345 (13.1)	158 (13.1)	93 (14.2)	94 (12.0)	<0.001
	Left colon	1595 (60.3)	769 (63.8)	346 (52.8)	480 (61.3)	
	Right colon	697 (26.4)	275 (22.8)	214 (32.7)	208 (26.6)	
BSG 2020 risk index procedure	Low	1360 (51.5)	702 (58.3)	235 (35.9)	423 (54.0)	<0.001
	High	1283 (48.5)	503 (41.7)	420 (64.1)	360 (46.0)	

Values are *n* (%) unless otherwise indicated. SIMD, Scottish Index of Multiple Deprivation; COPD, chronic obstructive pulmonary disease; PUD, peptic ulcer disease; ACE-I, angiotensin converting enzyme inhibitor; ARB, angiotensin receptor blocker; NSAIDs, non-steroidal anti-inflammatory drugs; HGD, high-grade dysplasia; BSG, British Society of Gastroenterology.

*Applies to the most advanced polyp if multiple polyps removed during the index procedure.

### Multivariate polynomial regression

Based on the initial univariable comparisons, the following variables were taken forward to multivariate polynomial regression analysis: age, sex, aspirin, statin, index advanced polyp, polyp number, polyp location, villous features, and BSG 2020 risk score. Index polyp number (OR 1.15 (95 per cent c.i. 1.07 to 1.25); *P* < 0.001) and index villous features (OR 1.49 (95 per cent c.i. 1.05 to 2.10); *P* = 0.025) were independently associated with the detection of advanced metachronous lesions within 2 years of the index polypectomy. No variable was independently associated with advanced lesions after 2 years (*Table 3*).

**Table 3:** Multivariate multinomial logistic regression of factors relating to metachronous lesion advancement and time after the index polypectomy

		Advanced polyp or CRC ≤2 years after the index polypectomy		Advanced polyp or CRC >2 years after the index polypectomy	
		OR (95% c.i.)	*P*	OR (95% c.i.)	*P*
Age	Years	1.01 (0.99,1.04)	0.369	1.01 (0.99,1.03)	0.551
Sex	Female	0.90 (0.63,1.30)	0.587	0.85 (0.61,1.19)	0.344
Aspirin	Yes	1.13 (0.76,1.69)	0.543	0.99 (0.67,1.46)	0.948
Statin	Yes	1.38 (0.94,2.03)	0.105	0.93 (0.64,1.33)	0.671
Index advanced	Yes	1.21 (0.75,1.97)	0.433	1.03 (0.68,1.56)	0.900
Index polyp number	Number	1.15 (1.07,1.25)	<0.001	1.02 (0.93,1.13)	0.676
Index location	Rectum	1.0		1.0	
	Left colon	0.76 (0.46,1.26)	0.290	0.88 (0.57,1.37)	0.577
	Right colon	1.61 (0.95,2.73)	0.079	0.91 (0.55,1.51)	0.718
Index villous features	Yes	1.49 (1.05,2.10)	0.025	1.03 (0.75,1.42)	0.847
Index BSG 2020	High	1.43 (0.88,2.33)	0.153	0.86 (0.55,1.34)	0.498

CRC, colorectal cancer; OR, odds ratio; BSG, British Society of Gastroenterology.

### British Society of Gastroenterology 2020 high- *versus* low-risk patients

Finally, a comparison was made regarding outcome between patients who would be deemed low risk (1360 patients) and high risk (1283 patients) based on their index screening colonoscopy findings, according to the current BSG 2020 guidelines^5^ (*Table 4*). There was a higher rate of both non-advanced metachronous polyps (44.4 *versus* 35.4 per cent) and advanced metachronous polyps (15.7 *versus* 11.8 per cent) amongst the BSG 2020 high-risk patients as compared with the low-risk patients (*P* < 0.001), but a similar rate of CRC (0.6 *versus* 1.2 per cent). The group who would be high risk per BSG 2020 criteria contained 503 (39.2 per cent) patients with no metachronous lesions, whereas the proportion with advanced polyps or CRC in the group who would be low risk was 13.0 per cent (177 patients). Comparable differences were observed when examining early metachronous lesions only (detected less than or equal to 2 years after the index colonoscopy): non-advanced polyps (23.9 *versus* 12.6 per cent for the high-risk group and the low-risk group respectively), advanced metachronous polyps (8.7 *versus* 4.3 per cent for the high-risk group and the low-risk group respectively), and CRC (0.2 *versus* 0.3 per cent for the high-risk group and the low-risk group respectively) (*P* < 0.001). However, when examining only those lesions detected greater than 2 years after the index colonoscopy, no significant differences were observed: non-advanced polyps (20.6 *versus* 22.7 per cent for the high-risk group and the low-risk group respectively), advanced metachronous polyps (7.1 *versus* 7.5 per cent for the high-risk group and the low-risk group respectively), and CRC (0.4 *versus* 0.9 per cent for the high-risk group and the low-risk group respectively) (*P* = 0.140).

**Table 4:** Factors associated with index BSG 2020 risk category

			Index BSG 2020 risk group		
			Low (total = 1360)	High (total = 1283)	*P*
No metachronous lesion			702 (51.6)	503 (39.2)	
Metachronous polyp/CRC	All	Non-advanced polyp	481 (35.4)	570 (44.4)	<0.001
		Advanced polyp	161 (11.8)	202 (15.7)	
		CRC	16 (1.2)	8 (0.6)	
	Early (≤2 years)	Non-advanced polyp	172 (12.6)	306 (23.9)	<0.001
		Advanced polyp	59 (4.3)	111 (8.7)	
		CRC	4 (0.3)	3 (0.2)	
	Late (>2 years)	Non-advanced polyp	309 (22.7)	264 (20.6)	0.140
		Advanced	102 (7.5)	91 (7.1)	
		CRC	12 (0.9)	5 (0.4)	

Values are *n* (%). BSG, British Society of Gastroenterology; CRC, colorectal cancer.

## Discussion

This study examined the impact of applying the current BSG 2020 surveillance guidelines to a large retrospective cohort of patients whose surveillance strategy after screening polypectomy was determined by previous guidance^17,18^. It was observed that the overall rate of metachronous advanced polyps or CRC was relatively low (14.6 per cent) and nearly half had no metachronous lesion. When patients who would still be recommended surveillance based on current BSG guidance were selected, the rate of advanced metachronous polyps or CRC remained low (16.3 per cent) and the rate of no metachronous lesion remained high (39.2 per cent). The rate of advanced metachronous polyps or CRC in the low-risk BSG 2020 patients was only marginally lower (13.0 per cent) and perhaps suggests that current protocols would benefit from refinement. Furthermore, while BSG 2020 risk stratification group was associated with a significant difference in overall metachronous lesion rate, it did not differentiate advanced and non-advanced metachronous lesions and was not significantly associated with late metachronous lesions detected after 2 years from the index polypectomy.

The BSG 2020 guidelines include a comprehensive literature review on which their recommendations are based^5^. Evidence is presented of a heightened risk of advanced adenoma/neoplasia at surveillance colonoscopy, with index findings of HGD^19–25^, increased polyp number^19,20,22,23,26–30^, and larger index polyps (predominantly greater than or equal to 20 mm)^19,20,22–26,29,31^. However, other studies that failed to find significant associations between metachronous advanced adenoma/neoplasia and index HGD^26,28,29,31–33^, polyp number^33–37^, or index polyp size at a lower threshold of greater than or equal to 10 mm are also highlighted^23^. In the current study, the presence of HGD or index polyp size greater than or equal to 10 mm did not correlate with advanced or non-advanced metachronous lesions. Increased index polyp number correlated with both advanced and non-advanced lesions and with the development of any metachronous lesion less than or equal to 2 years after the index colonoscopy. Additionally, index polyp number was an independent factor of early advanced metachronous lesions on multivariate polynomial regression. This may reflect a strong patient propensity to develop multiple colorectal polyps and/or incomplete polyp clearance at the index colonoscopy. A further factor found to independently correlate with early advanced metachronous lesions was villous index morphology. While BSG 2020 highlighted numerous studies associating villous morphology with metachronous advanced lesion risk^19,20,22,23,26,28,29,31,33,34,38^, it has historically not been included in UK-based risk stratification due to concerns over heterogeneity in pathological reporting and the additional surveillance workload that inclusion may produce^5^. However, this study suggests it may be a more useful basic pathological variable than others currently in use.

Other studies have examined the efficacy of BSG 2020 risk. In a retrospective study of more than 21 000 patients who underwent polypectomy, CRC incidence in BSG 2020 low-risk patients was significantly lower than in the general population without surveillance, suggesting that benefit from polyp clearance has already been derived and no further surveillance is required. CRC incidence in BSG 2020 high-risk patients was significantly higher than in the general population without surveillance and there was no significant difference with surveillance, perhaps suggesting benefit^39^. In contrast, this smaller, but more recent, study suggests that many BSG 2020 high-risk patients do not develop metachronous lesions and the rate of advanced metachronous lesions is only marginally higher than for low-risk patients. Additionally, although BSG 2020 high risk was associated with metachronous advanced lesions, when adjusting for potential confounders with multivariate analysis, it was not correlated with metachronous advanced lesions detected less than or equal to 2 years or greater than 2 years after index colonoscopy.

The current study suggests that risk stratification may benefit from refinement. The INCISE collaborative intends to evaluate the addition of a panel of novel risk factors for metachronous lesion development to the BSG 2020 risk score. Factors such as patient characteristics, protein expression, and genomic and transcriptomic features of index polyp tissue will be used, with the hope of increasing the positive yield of surveillance colonoscopy and reducing unnecessary invasive investigation for those at lower risk. A systematic review published by INCISE identified 49 gene mutations, single nucleotide polymorphisms, or haplotypes in 23 different genes/chromosomal regions (including KRAS, APC, EGFR, and COX1/2) that predicted metachronous adenoma or advanced adenoma development. Additionally, the expression of six proteins was found to be associated with metachronous adenoma (p53, β-catenin, COX2, Adnab-9, and ALDH1A1) or sessile serrated polyp (ANXA10) risk^40^. Most of the studies included in this review were relatively small and focused on a single biomarker. The INCISE project aims to integrate a wide range of novel risk factors using machine learning and produce a risk stratification tool to be delivered to clinicians.

The current study has a number of strengths. It is large and multicentred in nature. As patients were identified by interrogating both endoscopy- and pathology-based reporting software, a very low number of missed eligible patients would be anticipated during the study interval. A broad range of demographics, co-morbidities, medications, and index pathological characteristics were screened for impact on metachronous lesion risk. The majority of significant factors were carried forward to multivariate analysis to account for confounding and there was a sufficiently long follow-up. There are, however, limitations. It is retrospective and observational in nature and hence there was variability in the patient surveillance interval. While every effort was made to ensure follow-up colonoscopies were appropriate in each case, for example excluding those performed for polypectomy site checks, the exact indication was not always recorded and a proportion may have been performed due to further screening positivity or for symptomatic reasons, rather than representing true surveillance colonoscopies. Only those patients who participated in bowel screening, had a positive screening test, and proceeded to colonoscopy could be included. The mean screening uptake rate in NHS GG&C during the study interval was 51.7 per cent, the test positivity rate was 2.7 per cent, and the rate of patients with a positive screening test proceeding to colonoscopy was 76.2 per cent^41^. There is potential for selection bias at each stage in this process, whereby those who do not proceed are not represented. To date those patients who underwent polypectomy, but were not invited/did not return for surveillance were not included; however, a linked study examining such patients is underway. The surveillance protocols that were applied to the patients of this study are now historical and in general terms less conservative than current BSG 2020 guidance. Therefore, it may be anticipated that the burden of surveillance colonoscopy and rate of negative investigations has decreased. However, by using this patient group we were able to create a cohort of BSG 2020 high- and low-risk patients, who all underwent surveillance colonoscopy to allow for outcome analysis. Finally, in terms of CRC there was a low number of events (24). It has been highlighted previously that the finding of advanced metachronous polyps is only a surrogate marker for CRC risk^42^ and it is difficult to make firm conclusions about risk stratification of CRC.

In this study, BSG 2020 high-risk features were associated with metachronous lesion detection, particularly those detected less than or equal to 2 years after the index screening polypectomy. However, BSG 2020 risk grouping did not differentiate metachronous advanced and non-advanced lesions and was less discriminatory in lesions detected beyond 2 years. Additionally, the proportion of patients with no metachronous lesions in the BSG 2020 high-risk group (39.2 per cent) and the proportion with advanced polyps or CRC in the BSG 2020 low-risk group (13.0 per cent) were relatively high. This suggests that post-polypectomy surveillance may benefit from refinement and the INCISE project aims to do this by applying novel techniques to index pathology tissue and integrating relevant outputs to produce a valuable risk stratification tool than can be delivered to clinicians and patients.

## Data Availability

An anonymized, limited data set is available on the secure Glasgow Safe Haven platform.

## References

[1] Cancer Research UK . https://www.cancerresearchuk.org/health-professional/cancer-statistics/statistics-by-cancer-type/bowel-cancer (accessed May 2022)

[2] Pickhardt PJ , Pooler BD, Kim DH, Hassan C, Matkowskyj KA, Halberg RB. The natural history of colorectal polyps: overview of predictive static and dynamic features. Gastroenterol Clin North Am 2018;47:515–536 30115435 10.1016/j.gtc.2018.04.004 PMC6100796

[3] Simon K . Colorectal cancer development and advances in screening. Clin Interv Aging 2016;11:967–976 27486317 10.2147/CIA.S109285 PMC4958365

[4] Bonnington SN , Rutter MD. Surveillance of colonic polyps: are we getting it right? World J Gastroenterol 2016;22:1925–1934 26877600 10.3748/wjg.v22.i6.1925 PMC4726668

[5] Rutter MD , East J, Rees CJ, Cripps N, Docherty J, Dolwani S et al British Society of Gastroenterology/Association of Coloproctology of Great Britain and Ireland/Public Health England post-polypectomy and post-colorectal cancer resection surveillance guidelines. Gut 2020;69:201–223 31776230 10.1136/gutjnl-2019-319858 PMC6984062

[6] Hewitson P , Glasziou P, Irwig L, Towler B, Watson E. Screening for colorectal cancer using the faecal occult blood test, Hemoccult. Cochrane Database Syst Rev 2007; 2:CD001216. 10.1002/14651858.CD001216.pub2 PMC6769059 17253456

[7] McFerran E , O’Mahony JF, Fallis R, McVicar D, Zauber AG, Kee F. Evaluation of the effectiveness and cost-effectiveness of personalized surveillance after colorectal adenomatous polypectomy. Epidemiol Rev 2017;39:148–160 28402402 10.1093/epirev/mxx002 PMC5858033

[8] Hao Y , Wang Y, Qi M, He X, Zhu Y, Hong J. Risk factors for recurrent colorectal polyps. Gut Liver 2020;14:399–411 31547641 10.5009/gnl19097 PMC7366149

[9] Løberg M , Kalager M, Holme Ø, Hoff G, Adami HO, Bretthauer M. Long-term colorectal-cancer mortality after adenoma removal. N Engl J Med 2014;371:799–807 25162886 10.1056/NEJMoa1315870

[10] Richards PSM . *Report of the Independent Review of Adult Screening Programmes in England October 2019*

[11] *Integrated Technologies for Improved Polyp Surveillance—About INCISE* . https://www.gla.ac.uk/research/az/incise/aboutincise/ (accessed 15 February 2021)

[12] Mansouri D , McMillan DC, Grant Y, Crighton EM, Horgan PG. The impact of age, sex and socioeconomic deprivation on outcomes in a colorectal cancer screening programme. PLoS One 2013;8:e66063 10.1371/journal.pone.0066063 PMC3680425 23776606

[13] Fraser CG , Digby J, McDonald PJ, Strachan JA, Carey FA, Steele RJ. Experience with a two-tier reflex gFOBT/FIT strategy in a national bowel screening programme. J Med Screen 2012;19:8–13 22156144 10.1258/jms.2011.011098

[14] *Strengthening the Reporting of Observational Studies in Epidemiology* . https://www.strobe-statement.org/ (accessed November 2022)

[15] *Scottish Index of Multiple Deprivation* . https://www.gov.scot/collections/scottish-index-of-multiple-deprivation-2020/ (accessed 8 November 2021)

[16] Charlson ME , Pompei P, Ales KL, MacKenzie CR. A new method of classifying prognostic comorbidity in longitudinal studies: development and validation. J Chronic Dis 1987;40:373–383 3558716 10.1016/0021-9681(87)90171-8

[17] Atkin WS , Saunders BP. Surveillance guidelines after removal of colorectal adenomatous polyps. Gut 2002;51(Suppl 5):V6–V9 12221031 10.1136/gut.51.suppl_5.v6 PMC1867736

[18] Cairns SR , Scholefield JH, Steele RJ, Dunlop MG, Thomas HJ, Evans GD et al Guidelines for colorectal cancer screening and surveillance in moderate and high risk groups (update from 2002). Gut 2010;59:666–689 20427401 10.1136/gut.2009.179804

[19] Atkin W , Brenner A, Martin J, Wooldrage K, Shah U, Lucas F et al The clinical effectiveness of different surveillance strategies to prevent colorectal cancer in people with intermediate-grade colorectal adenomas: a retrospective cohort analysis, and psychological and economic evaluations. Health Technol Assess 2017;21:1–536 10.3310/hta21250 PMC5483643 28621643

[20] Atkin W , Wooldrage K, Brenner A, Martin J, Shah U, Perera S et al Adenoma surveillance and colorectal cancer incidence: a retrospective, multicentre, cohort study. Lancet Oncol 2017;18:823–834 28457708 10.1016/S1470-2045(17)30187-0 PMC5461371

[21] van Enckevort C , de Graaf A, Hollema H, Sluiter W, Kleibeuker J, Koornstra J. Predictors of colorectal neoplasia after polypectomy: based on initial and consecutive findings. Neth J Med 2014;72:139–145 24846927

[22] Fairley KJ , Li J, Komar M, Steigerwalt N, Erlich P. Predicting the risk of recurrent adenoma and incident colorectal cancer based on findings of the baseline colonoscopy. Clin Transl Gastroenterol 2014;5:e64 25472702 10.1038/ctg.2014.11 PMC4274367

[23] Huang Y , Gong W, Su B, Zhi F, Liu S, Bai Y et al Recurrence and surveillance of colorectal adenoma after polypectomy in a southern Chinese population. J Gastroenterol 2010;45:838–845 20336471 10.1007/s00535-010-0227-3

[24] Facciorusso A , Di Maso M, Serviddio G, Vendemiale G, Muscatiello N. Development and validation of a risk score for advanced colorectal adenoma recurrence after endoscopic resection. World J Gastroenterol 2016;22:6049–6056 27468196 10.3748/wjg.v22.i26.6049 PMC4948260

[25] Facciorusso A , Di Maso M, Serviddio G, Vendemiale G, Spada C, Costamagna G et al Factors associated with recurrence of advanced colorectal adenoma after endoscopic resection. Clin Gastroenterol Hepatol 2016;14:1148–1154.e4 27005802 10.1016/j.cgh.2016.03.017

[26] Martínez ME , Baron JA, Lieberman DA, Schatzkin A, Lanza E, Winawer SJ et al A pooled analysis of advanced colorectal neoplasia diagnoses after colonoscopic polypectomy. Gastroenterology 2009;136:832–841 19171141 10.1053/j.gastro.2008.12.007 PMC3685417

[27] Huang Y , Li X, Wang Z, Su B. Five-year risk of colorectal neoplasia after normal baseline colonoscopy in asymptomatic Chinese Mongolian over 50 years of age. Int J Colorectal Dis 2012;27:1651–1656 22763754 10.1007/s00384-012-1516-5

[28] Cubiella J , Carballo F, Portillo I, Quevedo JC, Salas D, Binefa G et al Incidence of advanced neoplasia during surveillance in high-and intermediate-risk groups of the European colorectal cancer screening guidelines. Endoscopy 2016;48:995–1002 27485482 10.1055/s-0042-112571

[29] van Heijningen EM , Lansdorp-Vogelaar I, Kuipers EJ, Dekker E, Lesterhuis W, Ter Borg F et al Features of adenoma and colonoscopy associated with recurrent colorectal neoplasia based on a large community-based study. Gastroenterology 2013;144:1410–1418 23499951 10.1053/j.gastro.2013.03.002

[30] Vemulapalli KC , Rex DK. Risk of advanced lesions at first follow-up colonoscopy in high-risk groups as defined by the United Kingdom post-polypectomy surveillance guideline: data from a single U.S. center. Gastrointest Endosc 2014;80:299–306 24796960 10.1016/j.gie.2014.02.1029

[31] Park SK , Kim NH, Jung YS, Kim WH, Eun CS, Ko BM et al Risk of developing advanced colorectal neoplasia after removing high-risk adenoma detected at index colonoscopy in young patients: a KASID study. J Gastroenterol Hepatol 2016;31:138–144 26404417 10.1111/jgh.13167

[32] Cubiella J , Carballo F, Portillo I, Cruzado Quevedo J, Salas D, Binefa G et al Incidence of advanced neoplasia during surveillance in high- and intermediate-risk groups of the European colorectal cancer screening guidelines. Endoscopy 2016;48:995–1002 27485482 10.1055/s-0042-112571

[33] Lee TJ , Nickerson C, Goddard AF, Rees CJ, McNally RJ, Rutter MD. Outcome of 12-month surveillance colonoscopy in high-risk patients in the National Health Service Bowel Cancer Screening Programme. Colorectal Dis 2013;15:e435–e442 23663559 10.1111/codi.12278

[34] Laiyemo AO , Murphy G, Albert PS, Sansbury LB, Wang Z, Cross AJ et al Postpolypectomy colonoscopy surveillance guidelines: predictive accuracy for advanced adenoma at 4 years. Ann Intern Med 2008;148:419–426 18347350 10.7326/0003-4819-148-6-200803180-00004

[35] Solakoğlu T , Köseoğlu H, Özer Sari S, Akin FE, Bolat AD, Yürekli ÖT et al Role of baseline adenoma characteristics for adenoma recurrence in patients with high-risk adenoma. Turk J Med Sci 2017;47:1416–1424 29151312 10.3906/sag-1502-105

[36] Lee JL , Cha JM, Lee HM, Jeon JW, Kwak MS, Yoon JY et al Determining the optimal surveillance interval after a colonoscopic polypectomy for the Korean population? Intest Res 2017;15:109–117 28239321 10.5217/ir.2017.15.1.109 PMC5323300

[37] Jung YS , Park DI, Kim WH, Eun CS, Park S-K, Ko BM et al Risk of advanced colorectal neoplasia according to the number of high-risk findings at index colonoscopy: a Korean Association for the Study of Intestinal Disease (KASID) study. Dig Dis Sci 2016;61:1661–1668 26809871 10.1007/s10620-016-4038-0

[38] Nusko G , Sachse R, Mansmann U, Wittekind C, Hahn EG. K-RAS-2 gene mutations as predictors of metachronous colorectal adenomas. Scand J Gastroenterol 1997;32:1035–1041 9361177 10.3109/00365529709011221

[39] Cross AJ , Robbins EC, Pack K, Stenson I, Patel B, Rutter MD et al Colorectal cancer risk following polypectomy in a multicentre, retrospective, cohort study: an evaluation of the 2020 UK post-polypectomy surveillance guidelines. Gut 2021;70:2307–2320 33674342 10.1136/gutjnl-2020-323411 PMC8588296

[40] Johnstone MS , Lynch G, Park J, McSorley S, Edwards J. Novel methods of risk stratifying patients for metachronous, pre-malignant colorectal polyps: a systematic review. Crit Rev Oncol Hematol 2021;164:103421 10.1016/j.critrevonc.2021.103421 34246774

[41] *NHS National Services Scotland, National Statistics: Scottish Bowel Screening Programme Statistics* . https://www.isdscotland.org/Health-Topics/Cancer/Publications/2011-08-30/2011-08-30-Bowel-Screening-Report.pdf (accessed November 2022)

[42] Lieberman D . Colon-polyp surveillance–do patients benefit? N Engl J Med 2014;371:860–861 25162893 10.1056/NEJMe1407152

